# Prophylactic antibiotics for the prevention of infection following operative vaginal delivery (ANODE): study protocol for a randomised controlled trial

**DOI:** 10.1186/s13063-018-2787-0

**Published:** 2018-07-24

**Authors:** Marian Knight, Linda Mottram, Shan Gray, Christopher Partlett, Ed Juszczak, the ANODE collaborative group, Marian Knight, Helen Enderby, Derek Tuffnell, Kim Hinshaw, Ranee Thakar, Abdul H. Sultan, Julia Sanders, Dharmintra Pasupathy, Philip Moore, James Gray, Oliver Rivero-Arias, Ed Juszczak, Louise Linsell, Aethele Khunda

**Affiliations:** National Perinatal Epidemiology Unit, Nuffield Department of Population Health, University of Oxford, Old Road Campus, Oxford, OX3 7LF UK

**Keywords:** Sepsis, Infection, Operative vaginal delivery, Prevention, Antibiotic prophylaxis, Randomised controlled trial

## Abstract

**Background:**

Sepsis is one of the most important causes of maternal death and severe morbidity worldwide. Studies conducted both in the UK and US have documented an additional risk associated with operative vaginal delivery. However, a Cochrane review, updated in 2017, identified only one small trial of prophylactic antibiotics following operative vaginal delivery, which included a total of 393 women. Given the small size of that trial, it recommended that further robust evidence is needed. Operative vaginal delivery rates vary worldwide, but typically 5–10% of women have operative vaginal births. A conservative estimated incidence of maternal infection following operative vaginal delivery is 4%, based on the one previous trial. There is, therefore, considerable scope for direct patient benefit from an effective preventive strategy.

**Methods/Design:**

This protocol describes a multicentre, randomised, blinded, placebo-controlled trial aiming to recruit 3424 participants from over 20 hospital sites in the UK. Women who have undergone an operative vaginal delivery at 36^+0^ weeks or greater gestation with no indication for ongoing antibiotics in the postpartum period and no contra-indications to prophylactic co-amoxiclav, will be randomised to receive a single intravenous dose of co-amoxiclav or placebo.

The primary outcome will be confirmed or suspected maternal infection within 6 weeks of delivery, as defined by one of (a) a new prescription of antibiotics for presumed perineal wound-related infection, endometritis or uterine infection, urinary tract infection with systemic features or other systemic infection, (b) systemic infection confirmed with a culture or (c) endometritis as defined by the US Centers for Disease Control and Prevention. Outcome information will be collected by a single telephone interview and questionnaire, with clinical data collected from medical records or the hospital laboratory if necessary, at 6 weeks post-delivery.

**Discussion:**

This randomised trial will investigate whether a prophylactic dose of antibiotic following operative vaginal delivery can reduce the incidence of infection and sepsis. If shown to be effective, this could lead to a change in recommended practice and the prevention of infection. Conversely, if there is no significant difference between the two arms, then this could contribute to a reduction in antibiotic use and improved antimicrobial stewardship.

**Trial registration:**

ISRCTN11166984. Registered on 23 September 2015.

**Electronic supplementary material:**

The online version of this article (10.1186/s13063-018-2787-0) contains supplementary material, which is available to authorized users.

## Background

Sepsis remains an important cause of maternal death and severe morbidity worldwide [1, 2]. For every woman who dies from sepsis, an estimated 50 women have severe sepsis (requiring level 2 or 3 critical care) but survive [3]. An increased risk of sepsis in association with caesarean section delivery has been recognised for many years [4], and UK National Institute for Health and Care Excellence (NICE) guidance recommends the use of prophylactic antibiotics at all caesarean deliveries [5], based on substantial randomised controlled trial evidence of effectiveness [6]. Studies conducted both in the UK and US have documented an additional risk associated with operative vaginal delivery [3, 7–9]. A Cochrane review identified only one small previous trial of prophylactic antibiotics following operative vaginal delivery, which included a total of 393 women, with a relative risk of 0.07 (95% confidence interval 0.00 to 1.21) for postpartum infection [10]. Given the small study size and extreme result, the World Health Organisation (WHO) recommends that prophylactic antibiotics following operative vaginal delivery should not be used routinely and that further robust evidence is needed [11].

Further work suggests that the burden of localised infection following operative vaginal delivery is also significant [12], with more than 10% of women experiencing symptoms of perineal wound infection in the 3 weeks following delivery. Women involved in prioritising childbirth-related perineal trauma outcomes have rated fear of perineal infection as the most important outcome they are concerned about in the first few weeks after childbirth-related perineal trauma [13].

Approximately 13% of women have an operative vaginal (forceps or ventouse) delivery in England. The associated infections could, therefore, represent a significant burden of potentially preventable morbidity [14]. Current national guidelines for intrapartum care make no reference to prophylactic antibiotics following instrumental delivery [15]. In common with the WHO, the UK Royal College of Obstetricians and Gynaecologists (RCOG) guidance on operative vaginal delivery [16] states that there are insufficient data to justify the use of prophylactic antibiotics. RCOG guidance on bacterial sepsis following pregnancy does not identify operative vaginal delivery as a risk factor for postpartum infection [17] and lack of awareness of the associated risk may contribute to a delay in diagnosis. Evidence suggests that progression to severe sepsis following delivery, particularly in association with group A streptococcal infection, can be very rapid [3]. This emphasises the importance of urgent investigation of potential prophylactic measures.

An estimated 104,000 women annually in the UK undergo forceps or ventouse deliveries [14]. The conservatively estimated incidence of maternal infection following operative vaginal delivery is 4%, based on the one previous trial [10], resulting in an estimated 4160 women potentially having an infection after instrumental delivery. Of these women, around 200 will be diagnosed with severe sepsis [7], and up to four may die from their infection [3]. There is, therefore, considerable scope for direct patient benefit from an effective preventive strategy.

### Trial objective

The primary objective of this multicentre, randomised, blinded, placebo-controlled trial is to investigate whether a single dose of prophylactic antibiotic following operative vaginal delivery is clinically effective for preventing confirmed or suspected maternal infection.

## Methods/Design

**Fig. 1:**
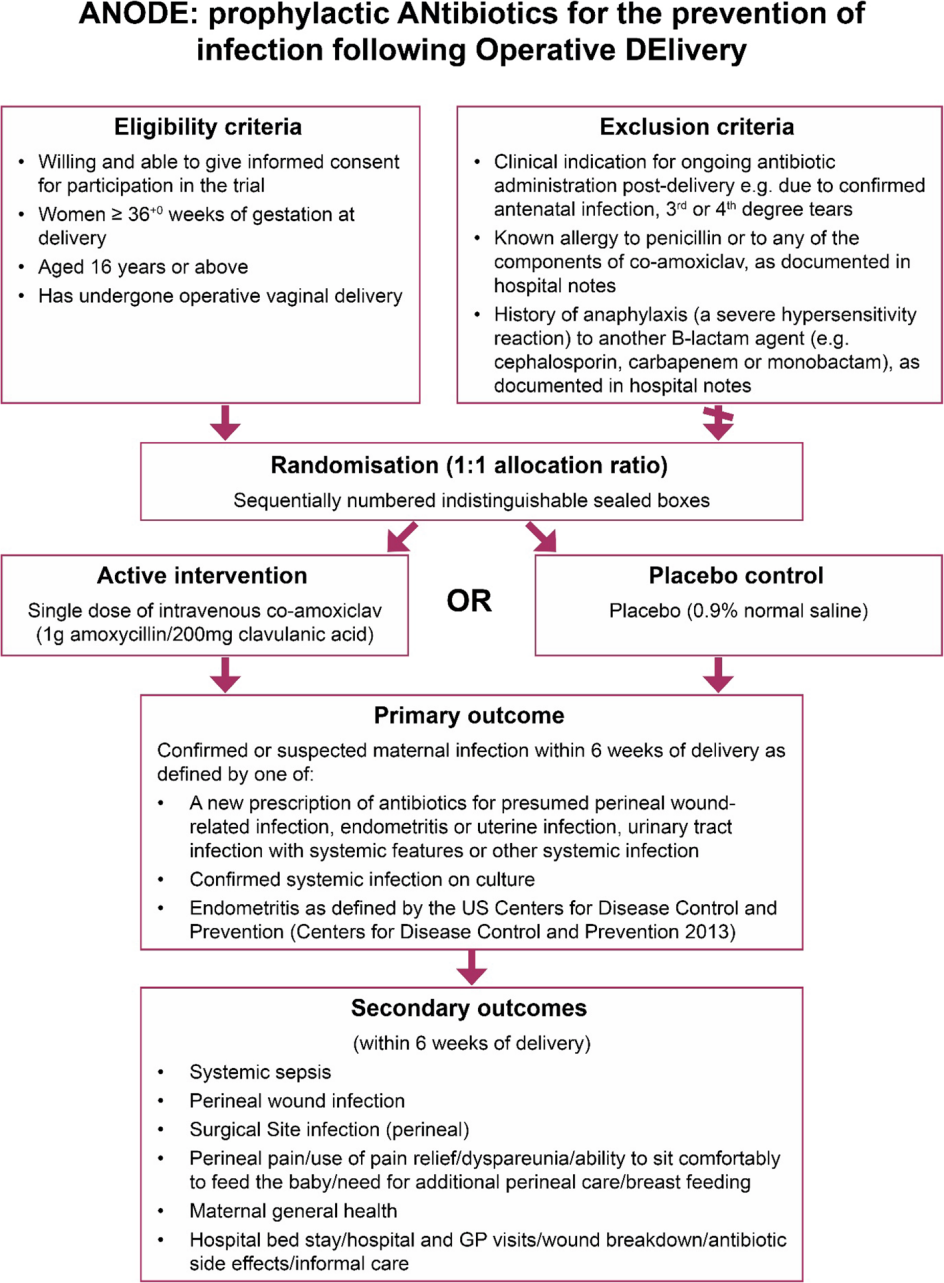
ANODE trial flow chart. GP general practitioner

The trial is an individually randomised trial of 3424 women who have undergone forceps or ventouse delivery at 36^+ 0^ weeks or greater gestation, with no indication for ongoing prescription of antibiotics in the postpartum period and no contra-indications to prophylactic co-amoxiclav. They will be randomised to receive a single intravenous dose of prophylactic co-amoxiclav (1 g amoxicillin plus 200 mg clavulanic acid) or placebo (intravenous saline) administered as soon as possible after delivery of the baby but no later than 6 hours after delivery. The trial design is summarised in Fig. 1. The SPIRIT checklist is provided in Additional file 1.

### Inclusion criteria

- Women aged 16 years or above, and willing and able to give informed consent.
- Women who have had an operative vaginal delivery at 36^+ 0^ weeks or greater gestation.

### Exclusion criteria

Women may not enter the trial if any of the following apply:

- Clinical indication for ongoing antibiotic administration post-delivery, e.g. due to confirmed antenatal infection, or third or fourth degree tears. Receiving antenatal antibiotics, for example for maternal group B streptococcal carriage or prolonged rupture of membranes, is not a reason for exclusion if there is no indication for ongoing antibiotic prescription post-delivery.
- Known allergy to penicillin or to any of the components of co-amoxiclav, as documented in their hospital notes.
- History of anaphylaxis to another β-lactam agent (for example cephalosporin, carbapenem or monobactam), as documented in their hospital notes.

### Primary outcome

The primary outcome is confirmed or suspected maternal infection within 6 weeks of delivery, as defined by one of:

- A new prescription of antibiotics for presumed perineal wound-related infection, endometritis or uterine infection, urinary tract infection with systemic features or other systemic infection.
- Systemic infection confirmed with a culture.
- Endometritis as defined by the US Centers for Disease Control and Prevention [18].

### Secondary outcomes

Secondary outcomes are assessed within 6 weeks of delivery and include:

- Systemic sepsis: defined according to modified systemic inflammatory response syndrome criteria for pregnancy used in previous population-based surveillance studies [3, 19].
- Perineal wound infection: defined according to the Public Health England surveillance definition of surgical site infection [20], classified as superficial incisional infection, deep incisional infection or organ/space infection.
- Perineal pain, use of pain relief, dyspareunia, ability to sit comfortably to feed the baby, need for additional perineal care, breastfeeding: identified using standard questions developed for the HOOP study [21] and the PREVIEW study [22].
- Maternal general health: As elicited by the EQ-5D-5L [23].
- Hospital bed stay, hospital and general practitioner visits, wound breakdown or antibiotic side effects: identified through specific questions included in the maternal questionnaire to include medications prescribed, critical care admission, hospital inpatient admissions, outpatient visits, and midwife and practice nurse visits. All side effects of the IMP (Investigational Medicinal Product) will be recorded.

### Trial procedures

#### Informed consent

Information about the trial will be widely available throughout the maternity unit and community clinics in the form of posters and leaflets. All women at participating centres will be provided with written information about the trial during their pregnancy, for example, at their antenatal booking visit, as part of their hand-held notes or at their 19–21 week ultrasound scan visit (centre-dependent).

On admission, all women in labour or admitted for induction will be reminded about the trial by their health-care professional. Information about the trial will be provided if not previously seen. After the clinical decision for operative vaginal delivery is made and the woman or her representative has given consent for an operative vaginal delivery, the woman will be approached by her midwife, obstetrician or anaesthetist to discuss the trial.

**Fig. 2:**
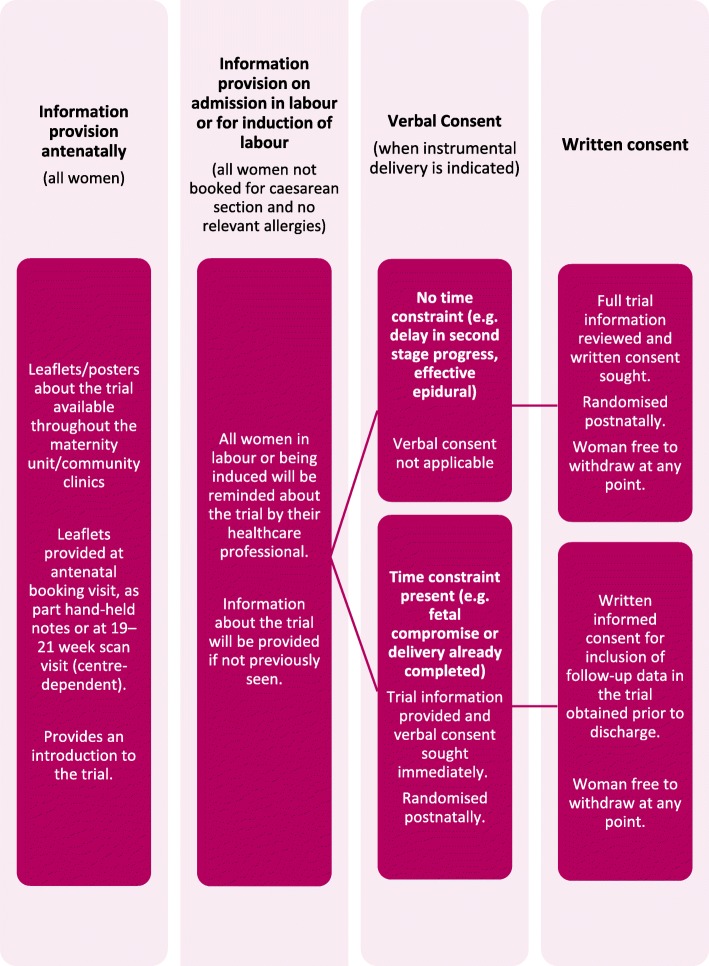
Consent and randomisation processes

The following approaches will be used by the woman’s midwife, obstetrician or anaesthetist to obtain informed consent, depending on the clinical circumstances (Fig. 2):

- Where there is no time constraint (such as for an operative vaginal delivery for delayed second-stage progress), the health-care professional will discuss the trial with the woman and provide her with the participant information leaflet. If she is happy to join the trial, written informed consent will be obtained.
- Where there is a time or other constraint (such as for an operative vaginal delivery for suspected fetal compromise or delivery is already completed), women will be approached to give verbal consent. It is possible that urgent deliveries are associated with a lower standard of asepsis, and so it is particularly important that these women are able to participate in the trial. If the attending obstetrician or midwife feels it is appropriate, the woman will be provided with verbal information about the trial and asked if she is willing to participate, in principle. If she agrees, she will be randomised. All women enrolled under this procedure will be approached before discharge by study midwives to give full written consent for inclusion of their data in the trial and for participation in the planned follow-up.

#### Randomisation and blinding

A randomisation list will be generated using permuted blocks of variable size to ensure balance and unpredictability overall. Centres will be supplied with sealed sequentially numbered indistinguishable packs containing the active drug or placebo (saline solution), as designated by the randomisation list. Women will be randomised by the allocation of the next sequentially numbered pack, once consent and eligibility are established.

The women, research midwives collecting outcome information and most clinicians will remain blind to allocation. The individuals responsible for preparing and checking the trial drug, who may be, for example, a doctor, midwife, nurse, operating department practitioner or other health-care professional (centre-dependent), will be the only individuals not blinded to allocation (these individuals will not be involved in the collection of outcome information).

#### Data collection

For eligible women, clinical details will be collected at trial entry (randomisation). This will include details to confirm eligibility and basic demographic, medical and obstetric details, including details of any antibiotic treatment in the 7 days before delivery.

Data will be collected at hospital discharge after delivery by extracting information from the woman’s clinical records by the research midwife, and at 6 weeks post-delivery by telephone interview with a research midwife to obtain information on the primary outcome, following which each woman will be sent a postal or online questionnaire (as preferred by each woman) for collection of data on secondary outcomes. Text reminders for completion will be sent as appropriate, with the option for telephone completion in the event of a delayed response to ensure a high response rate. Information about any hospital readmissions will be collected from hospital records by the research midwife.

To capture any additional related health outcomes after 6 weeks post-delivery, consent will be sought to extract hospital inpatient, critical care, outpatient and emergency department visit information from Hospital Episode Statistics or NHS Wales Informatics Service up to 1 year post randomisation for all trial participants.

**Fig. 3:**
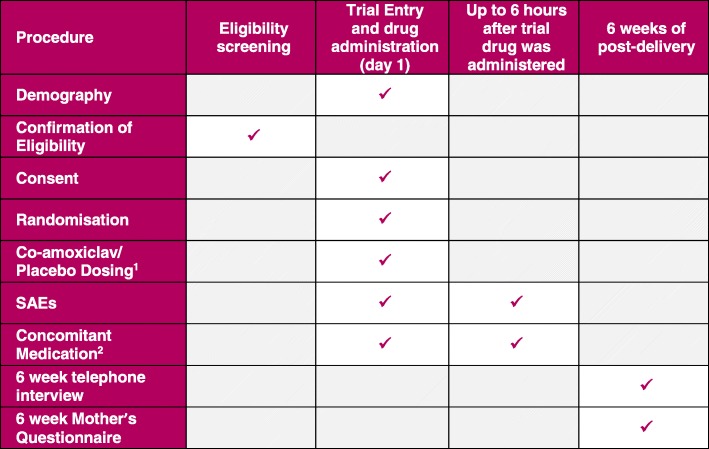
Trial procedures and assessments. SAE serious adverse event

The trial procedures are summarised in Fig. 3.

#### Safety reporting

The safety reporting window for this trial will be from administration of the intervention to 6 h post administration or discharge (whichever is sooner). The trial will follow the standard operating procedure for safety reporting of the National Perinatal Epidemiology Unit (NPEU) Clinical Trials Unit. Specific arrangements for this trial are summarised as follows.

##### Recording adverse events

Non-serious adverse events will not be routinely recorded as the intervention is a licensed product that is being given at a standard dose. However, adverse events that are relevant to the study outcomes will be recorded.

##### Reporting serious adverse events

All serious adverse events will be reported immediately, at least within 24 h, except the following serious adverse events, which are not considered to be causally related to the trial intervention:

- Birth defects or congenital anomalies
- Hypertensive disorders of pregnancy (e.g. pre-eclampsia or eclampsia)
- Postpartum haemorrhages with onset before the intervention

##### Expectedness

Expectedness will be determined according to the up-to-date Summary of Product Characteristics for co-amoxiclav.

### Sample size

The existing literature suggests a conservative estimate of the background rate of maternal infection following operative delivery of 4% [10]. We have assumed an estimated relative risk reduction of 50% in this rate to 2% in the treatment arm with antibiotics (the single trial relating to operative delivery suggests a greater reduction than this, but this rate of reduction is based on that seen in the more robust antibiotic prophylaxis for caesarean section trials [6]). To detect such a difference with 90% statistical power at the two-sided 5% level of significance requires 1626 per group. With an estimated 5% loss to follow-up, the trial requires 1712 per group, a total of 3424 women.

### Sites and site monitoring

The study will be conducted at 27 sites in the UK. Sites will be monitored according to the standard operating procedure for site monitoring of the NPEU Clinical Trials Unit, which includes at least one site visit over the course of the trial as well as triggered visits if required. Source data verification conducted over the course of the trial will be at least 1% and this will increase if a large number of discrepancies are found.

### Statistical analysis

A statistical analysis plan will be produced separately, prior to unblinding of data for the first interim analysis. This will be submitted for approval by the trial steering committee following review and comments from the data management committee.

Demographic and clinical data will be summarised with counts and percentages for categorical variables, means (with standard deviations) for normally distributed continuous variables and medians (with interquartile or simple ranges) for other continuous variables.

Women will be analysed in the groups to which they were randomly assigned, comparing the outcome of all women allocated to active treatment with all those allocated to placebo, regardless of deviation from the protocol or treatment received (referred to as the intention-to-treat population).

Interim analyses will be undertaken annually for review by the data management committee. The data management committee will inform the trial steering committee if, in its view, there is proof beyond all reasonable doubt that the trial should be terminated. The decision to inform the trial steering committee of such a finding will, in part, be based on statistical considerations. Appropriate proof beyond reasonable doubt cannot be specified precisely. A difference of at least 3 standard errors in the interim analysis of a major end point may be needed to justify halting or modifying the study prematurely.

For the main analyses, binary outcomes will be reported using unadjusted risk ratios, whilst normally distributed continuous outcomes will be analysed using a *t*-test and reported using unadjusted mean differences. For excessively skewed continuous outcomes, median differences will be presented instead. Moreover, 95% confidence intervals will be presented for the analysis of the primary outcome, while 99% confidence intervals will be presented for secondary outcomes. Two-sided statistical testing will be performed throughout.

Since randomisation is not stratified, the primary analysis will not be adjusted for other factors, but a sensitivity analysis will be conducted including centre as a random effect.

No subgroup analyses are planned and any sensitivity analyses requiring adjustment will be performed using log binomial regression for binary outcomes and linear regression for continuous outcomes.

Loss to follow-up is expected to be a maximum of 5% for short-term outcomes up to 6 weeks. A pre-specified sensitivity analysis will be undertaken, examining the primary outcome restricted to women who had not received antibiotics in the 7 days prior to delivery, in case any masking of a prophylactic effect is occurring by inclusion of pre-treated women.

Missing data as a result of women being lost to follow-up is expected to be minimal. All reasonable efforts will be taken to minimise loss to follow-up, which is expected to be no more than 5%. Women for whom no follow-up primary outcome data are received will be compared to women with data on demographic and clinical characteristics to assess any potential bias due to the impact of the missing data. As there is expected to be a link between outcome and loss to follow-up, imputation techniques will not provide any meaningful information and will not be used.

## Discussion

This randomised controlled trial is responding to WHO and Cochrane review recommendations that further robust evidence is needed in relation to operative vaginal delivery and the use of prophylactic antibiotics to reduce rates of sepsis and infection [10, 11]. With the latest figures showing that approximately 13% of women have an operative vaginal delivery in England, this equates to a significant burden of potentially preventable morbidity [14]. The RCOG guidance on operative vaginal delivery [16] also states that there are insufficient data to justify the use of prophylactic antibiotics in operative vaginal delivery, referencing the Cochrane review [10]. Thus, it is clear that there is a real need to determine the effectiveness of prophylactic antibiotics in the prevention of infection and sepsis following operative vaginal delivery so that this important question may be answered and recommendations made to guide clinical practice.

Additionally, there are widespread concerns about antimicrobial stewardship and the need to preserve the utility of a potentially limited resource [24]. The use of prophylactic antibiotics after operative vaginal delivery is widespread, despite the clear lack of evidence and WHO recommendation that they should not be used. If this trial shows that antibiotic prophylaxis is ineffective in the prevention of infection and sepsis following operative vaginal delivery, it may provide an important driver to reduce inappropriate antibiotic use and thus, help safeguard against increasing pathogen resistance.

### Trial status

At the time of manuscript submission, the trial is still currently recruiting participants.

## Additional file

Additional file 1: SPIRIT 2013 Checklist: Recommended items to address in a clinical trial protocol and related documents. (DOC 120 kb)
